# Advantages of time-dependent diffusion MRI for quantitative microstructural mapping in breast tumors

**DOI:** 10.3389/fonc.2025.1537529

**Published:** 2025-03-25

**Authors:** Lei Bao, Sijie Li, Zhuo Wang, Yang Sun, Ying Qiu, Zhiwei Shen, Xiaoxiao Zhang, Xue Chen, Xiaoxiao Zhang, Junyu Zhang, Tiefeng Ji

**Affiliations:** ^1^ The First Hospital of Jilin University, Department of Radiology, Changchun, Jilin, China; ^2^ The First Hospital of Jilin University, Department of Breast Surgery, Changchun, Jilin, China; ^3^ Department of Clinical, Philips Healthcare, Beijing, China

**Keywords:** time-dependent diffusion MRI, breast tumor, microstructural characteristics, ADC, diagnostic efficacy

## Abstract

**Objectives:**

Time-dependent diffusion MRI (TD-MRI) can measure tumor tissue microstructure, but its effectiveness in differentiating benign from malignant breast tumors is unclear. This study aims to investigate the diagnostic value of TD-MRI microstructural features for distinguishing between benign and malignant breast tumors.

**Methods:**

This prospective study included 44 patients with malignant breast tumors and 28 with benign tumors. All subjects underwent the IMPULSED protocol on a 3.0-T MRI scanner. Imaging data were analyzed using least squares fitting in MATLAB, yielding Dex (extracellular diffusivity), Vin (intracellular volume fraction), Dmean (cell diameter), Vin/Dmean, and ADC values. The molecular subtypes of breast cancer are classified based on immunohistochemistry (IHC) results.

**Results:**

Malignant tumors exhibited significantly lower Dmean (17.37 ± 2.74 µm *vs.* 22.47 ± 3.85µm, p<0.0001), higher Vin (0.41 ± 0.13% *vs.* 0.19 ± 0.10%, p<0.0001), and higher Vin/Dmean (2.13 ± 0.66 *vs.* 0.93 ± 0.61, p<0.0001) compared to benign tumors. No significant difference was found in Dex (2.15 ± 0.28 um^2^/ms *vs.* 2.25 ± 0.31 um^2^/ms, p>0.05). Strong correlations were observed: positive between ADC and Dmean, and negative between ADC and both Vin and Vin/Dmean. AUC values for Vin (0.92; 95% CI: 0.86-0.99), and Vin/Dmean (0.91; 95% CI: 0.83-0.98) surpassed those for ADC.

**Conclusion:**

TD-MRI microstructure mapping effectively differentiates benign from malignant breast tumors, highlighting its potential to improve diagnostic accuracy for lesions.

## Introduction

By 2020, Breast Cancer has become the most prevalent malignant tumors among women, with a persistently high mortality rate (1, 2). However, there is a lack of highly specific noninvasive indicators to accurately distinguish benign and malignant breast lesions. The cell diameter and density in malignant breast tumors are typically significantly different from those in benign tumors (3). Additionally, cell size plays an important role in assessing cellular functions such as metabolism, proliferation, and tissue growth, making it relevant to the diagnosis and treatment of diseases (4–6).

Quantitative methods for noninvasively measuring cell size *in vivo* remains lacking. Traditional microscopic methods, such as microscopes, cell counters, and flow cytometry, rely on invasive sampling, which could involve sampling errors and observer bias. In clinical practice, the apparent diffusion coefficient (ADC) is widely used to differentiate between benign and malignant breast tumors (7, 8), but it fails to quantify cell size (9). Time-dependent diffusion-weighted magnetic resonance imaging (TD-MRI) utilizes various diffusion weightings (q-space) and diffusion times (t-space) to provide detailed tissue microstructure information, such as cell size and cell volume fraction at different diffusion length scales (9–11). Incorporating Oscillating Gradient Spin Echo (OGSE) into TD-MRI enhances sensitivity to intracellular diffusion by achieving shorter diffusion times, minimizing the influence of water exchange across cell membranes (12, 13). This method offers a clear advantage over using PGSE alone in TD-MRI (10, 14).

Several researchers have attempted to use TD-MRI to measure microstructural data of tumor tissues, including methods like DDR (15), VERDICT (3, 16), and POMACE (17). However, these methods have limitations, such as prolonged scan times due to extended diffusion times or neglecting microstructural features like cell diameter and intracellular volume fraction. TD-MRI methods based on Pulse Gradient Spin Echo (PGSE) and Oscillating Gradient Spin Echo (OGSE) have been applied in diseases such as prostate cancer and gliomas (15, 18–20) with VERDICT, POMACE and IMPULSED methods. Xu’s application of the IMPULSED (8, 21) (Imaging Microstructural Parameters Using Limited Spectrally Edited Diffusion) model introduced OGSE to measure the microstructural parameters of human breast tumors, demonstrating OGSE’s capability to directly measure cellular- level microstructural characteristics, thus providing valuable insights for tumor development and treatment response assessment. However, the study was limited to a small number of clinical samples and a lack of comparison with clinical indicators.

Therefore, our study aims to use microstructural parameters derived from TD-MRI to explore the relationship between the microstructures of benign and malignant breast tumors, compare the diagnostic efficacy of microstructural parameters and ADC, and investigate the differences in microstructural parameters of malignant breast tumors across distinct molecular subtypes (such as Luminal A, Luminal B, Basal-like, HER2-enriched).

## Materials and methods

### Patient inclusion criteria

The prospective study was approved by the Ethics Committee of our hospital (24K286-001), with informed consents obtained from all participants. 145 patients who met the inclusion criteria were enrolled between July 2023 and June 2024. The inclusion criteria were: 1) histologically confirmed primary breast cancer or benign breast tumors; 2) availability of clinical and complete pathological data; 3) conventional breast MRI and TD-MRI scans performed within one week before surgery or biopsy; 4) lesion diameter of ≥10 mm; 5) no prior surgical resection, neoadjuvant chemotherapy, or other treatments following breast tumor discovery. The exclusion criteria were: 1) unresectable tumors(n=5); 2) prior breast tumor surgery or other treatments before the breast MRI examination(n=4); 3) Incomplete clinical or medical records(n=12); 4) image quality insufficient for diagnostic needs(n=3); 5) allergies to MRI contrast agents or inability to cooperate with the examination(n=4); 6) No breast MRI examination(n=45). Finally, 44 cases of malignant tumors and 28 cases of benign tumors were included with complete histopathological data from immunohistochemical staining (IHC) conducted on pathology slides.

### MRI data acquisition

The TD MRI technique requires acquiring diffusion MRI signals at varying diffusion times to capture the diffusion time-dependence in different microstructural components, therefore measuring diffusion within solid tumors. All scans were performed using a 3.0-T MRI scanner (Ingenia Elition, Philips Healthcare, the Netherlands) with a maximum gradient of 45 mT/m and maximum slew rate of 220 mT/m/ms, using a 16-channel breast array coil with the participant in the prone position.

**Figure 1:**
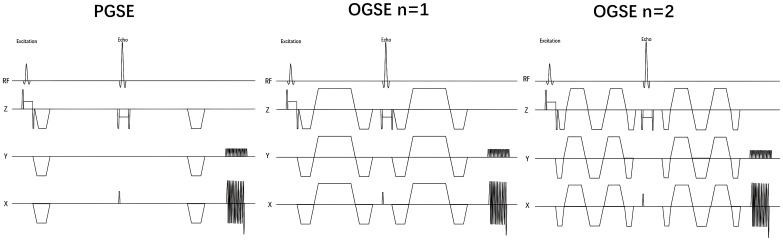
Using the IMPULSED method to acquire pulse diagrams of Pulsed Gradient Spin–Echo and Oscillating Gradient Spin–Echo in the breast. Time–dependent diffusion MRI signals depend on the diffusion time (td). The diffusivity of water molecules within a cellular environment is influenced by this diffusion time. By using PGSE and OGSE diffusion encoding schemes at different diffusion times, measurements can be made to help reconstruct microstructural properties using biophysical models.

**Figure 1** shows the Time-dependent diffusion MRI (TD-MRI) using a combination of the Pulsed Gradient Spin Echo (PGSE) sequence and the Oscillating Gradient Spin Echo (OGSE) sequence, with different diffusion times, to acquire MRI diffusion signals and determine the diffusion time dependence of various microstructural parameters.

After routine MRI scans, which include T1-weighted imaging, T2-weighted imaging, fat-suppressed T2-weighted imaging, and diffusion-weighted imaging (DWI), an OGSE diffusion MRI sequence combining OGSE and PGSE was implemented with trapezoid-cosine gradients and echo-planar imaging acquisition. The positioning of OGSE and PGSE scans was based on fat-suppressed T2-weighted imaging to cover the area where the breast tumor has the largest diameter. OGSE data were acquired at OGSE N2 (33Hz, duration of diffusion gradient = 63.9 ms, two cycles, b = 0, 100, 200, 260 sec/mm^2^) and OGSE N1 (17Hz, duration of diffusion gradient = 63.9 ms, one cycle, b = 0, 250, 500, 750, 1000 sec/mm^2^), and PGSE at a diffusion duration and separation of 15.9 and 117.1 ms, respectively (b = 0, 300, 600, 900, 1200, 1500 sec/mm^2^). The following parameters were used for both sequences: three diffusion directions; repetition time ms/echo time ms, 3000/143; field of view, 160 × 160 mm; in-plane resolution, 2.75 × 2.75 mm; number of slices, 5; and section thickness, 5 mm (**Table 1**). Finally, enhanced T1-weighted images were obtained.

**Table 1:** Sequence parameters for OGSE and PGSE.

Sequence	δ/△ (ms)	f (Hz)	b value (s/mm2)	Gmax(mT/m)	TR(ms)	TE(ms)	FOV(mm)	IPR(mm)	Slice Thickness (mm)	Scan duration (minute)
**PGSE**	15.9/117.1	0	0,300,600,900,1200,1500	45	3000	143	160×160	2.75×2.75	5	3:45
**OGSE N1**	63.9/71.7	17	0,250,500,750,1000	29.95	3000	143	160×160	2.75×2.75	5	2:33
**OGSE N2**	63.9/71.7	33	0,100,200,260	30.69	3000	143	160×160	2.75×2.75	5	1:03
**T1WI**	\	\	\	\	565	13	280×340	1.0×1.0	48	2:38
**T2WI**	\	\	\	\	4655	70	280×340	1.0×1.0	48	3:15

TD – MRI (OGSE and PGSE) with the IMPULSED protocol and conventional MRI parameters.

This study combines PGSE and OGSE to cover a range of diffusion times from short to long (63.9 ms to 117.1 ms). While OGSE with optimized gradient oscillations achieves a shorter effective diffusion time (Δ= 63.9 ms) by balancing total encoding duration (T) and oscillation parameters, it enhances sensitivity to restricted diffusion in smaller cellular compartments (8, 22). In contrast, PGSE extends the coverage to longer diffusion times (Δ=117.1 ms), capturing slower extracellular water mobility. Based on the IMPULSED model, low b-values (0–260 sec/mm^2^) primarily characterize extracellular diffusion (Dex), whereas higher b-values (up to 1500 sec/mm^2^) amplify sensitivity to intracellular diffusion, leveraging the pronounced signal attenuation from restricted water motion in confined spaces (23).

### Image analysis

All images were transformed from DICOM format to NIFTI format using MRIcroGL software (https://www.nitrc.org/projects/mricrogl/). The region of interest (ROI) on the largest plane of the breast tumor based on OGSE was delineated using ITK-SNAP software (version 3.6, http://www.itksnap.org) to generate a mask image. The ROI excludes tumor necrotic areas and blood vessels, as determined by two radiologists with 5 years of experience in diagnostic imaging, who were blinded to the diagnostic outcome, with reference to contrast-enhanced MRI breast images. Using the IMPULSED model for post-processing of raw data, TD-MRI can provide microstructural information of the lesions. Fitting was performed using the least squares curve fitting in MATLAB software (version 2022b, https://www.mathworks.com/). Dex (unit:µm²/ms), Vin (unit:%), Dmean (unit:µm), and Vin/Dmean (reflecting the percentage of microstructure and cell density) were obtained. The ADC values were obtained by performing log-linear fitting of all b values at each diffusion time (td). The parameters were constrained based on physiologically relevant values, specifically: 0 < Dmean< 30 μm, 0 < Vin < 1, and 0 < Dex < 3.5 μm²/ms.

### Histopathological information

Tissue sections obtained from surgical resection or biopsy of malignant breast tumors undergo immunohistochemical (IHC) staining to assess estrogen receptor (ER) and progesterone receptor (PR) status (defined as the percentage of positively stained tumor nuclei), human epidermal growth factor receptor 2 (HER2) status, Ki-67 proliferation index, and lymph node metastasis (LN) presence. Patients are classified into molecular subtypes (Luminal A, Luminal B, Basal-like, and HER2-enriched) based on IHC results (24). For Her-2 status, tumors are classified as Her-2 negative if IHC staining is 0 or 1+, and Her-2 positive if staining is 3+ (25). Tumors with IHC staining of 2+ require further confirmation with fluorescence *in situ* hybridization (FISH): non-amplified FISH results indicate Her-2 negativity, whereas amplified FISH results indicate Her-2 positivity (25).

### Statistical analysis

Statistical analyses were performed using GraphPad Prism software (version 9.5; www.graphpad.com/scientific-software/prism/). Normality of distribution was assessed through the Kolmogorov-Smirnov test for sample sizes >50 and the Shapiro-Wilk test for n ≤ 50. For comparisons of microstructural parameters between benign and malignant breast tumors, parametric data were analyzed with unpaired two-tailed t-tests, while non-parametric data were evaluated using Mann-Whitney-U tests. To compare the differences in time-varying diffusion MRI microstructural parameters among subtypes, a one-way analysis of variance (ANOVA) was performed. Bivariate correlations between microstructural parameters and apparent diffusion coefficient (ADC) values were examined using Pearson’s correlation coefficient for normally distributed variables and Spearman’s rank correlation coefficient for non-normally distributed data. Diagnostic performance was evaluated through receiver operating characteristic (ROC) curve analysis, with the area under the curve (AUC) reported alongside 95% confidence intervals (CI), Sensitivity and Specificity. Statistical significance was defined as p < 0.05 throughout all analyzes.

## Result

The baseline and clinical information of all patients with breast tumors are summarized in **Table 2**. In the benign tumor group, surgical pathology revealed as breast fibroadenoma (n=15), fibroadenomatous hyperplasia (n=8), and intraductal papilloma (n=5). In the malignant tumor group, there are ductal carcinoma *in situ* (n=4), invasive ductal carcinoma (n=24), invasive lobular carcinoma (n=13), and papillary carcinoma (n=3). None of the patients in the malignant tumor group received any prior treatments (including radiotherapy, chemotherapy, or surgery), and histopathological data were obtained through surgical excision or biopsy.

**Table 2:** Baseline participant and tumor characteristics.

Characteristics	Malignant breast tumor(n=44)	Benign breast tumor(n=28)
Age(year)	49.84±11.26	37.36±10.17
Menstruation state		
Premenopausal women	24 (54.5%)	25 (89.3%)
Postmenopausal women	20 (45.5%)	3 (10.7%)
BI–RADS classification		
BI–RADS III	3 (6.8%)	17 (60.7%)
BI–RADS IVa	3 (6.8%)	11 (39.3%)
BI–RADS IVb	12 (27.3%)	
BI–RADS IVc	7 (15.9%)	
BI–RADS V	11 (25.0%)	
BI–RADS VI	8 (18.2%)	
Tumor diameter (cm)	2.58±1.34	1.66±1.10

Baseline characteristics of malignant tumors of the breast (n=44)		
IHC Characteristics		
	Positive	Negative
ER	33 (75.0%)	11 (25.0%)
PR	29 (65.9%)	15 (34.1%)
Ki–67	28 (63.6%)	16 (36.4%)
Her–2	10 (22.7%)	34 (77.3%)
Lymph nodes status	28 (63.6%)	16 (36.4%)
Cancer subtype	Luminal A:14	
	Luminal B:12	
	Basal–like:10	
	HER2–enriched:8	
Pathohistological grading	I:4 (9.1%)	
	II:27 (61.4%)	
	III:13 (29.5%)	
Clinical stage	Stage 0 :2 (4.5%)	
	StageI:10 (22.7%)	
	StageII:20 (45.5%)	
	Stage III:9 (20.5%)	
	Stage IV:3 (6.8%)	

ER, estrogen receptor; PR, progesterone receptor; HER2, human epidermal growth factor receptor 2.

BI–RADS Categories: III (Probably Benign), IVa (Low Suspicion for Malignancy), IVb (Moderate Suspicion for Malignancy), IVc (High Suspicion for Malignancy), V (Highly Suggestive of Malignancy), VI (Known Biopsy–Proven Malignancy). Comparison of baseline information for benign and malignant breast tumors.

**Figure 2:**
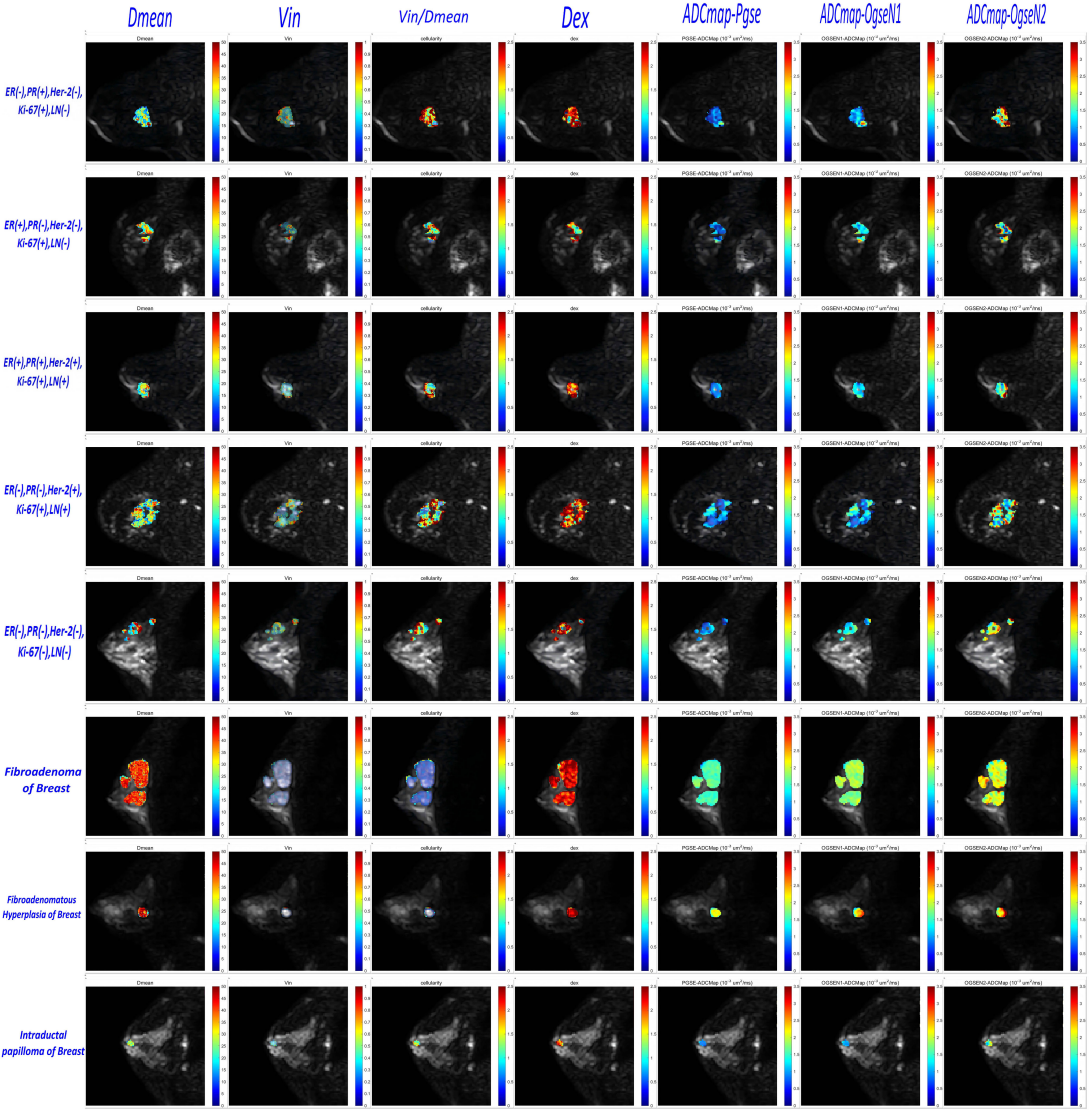
Microstructural characterization of benign and malignant breast tumors in which malignant breast tumors include five different receptor status and LN status categories and benign breast tumors include three different pathohistological types. (TD–MRI technique requires acquisition of diffusion MRI signals at varying diffusion times by using a combination of oscillating gradient spin–echo (OGSE) and pulsed gradient spin–echo (PGSE) sequences to measure diffusion within solid tumors.).

**Figure 3:**
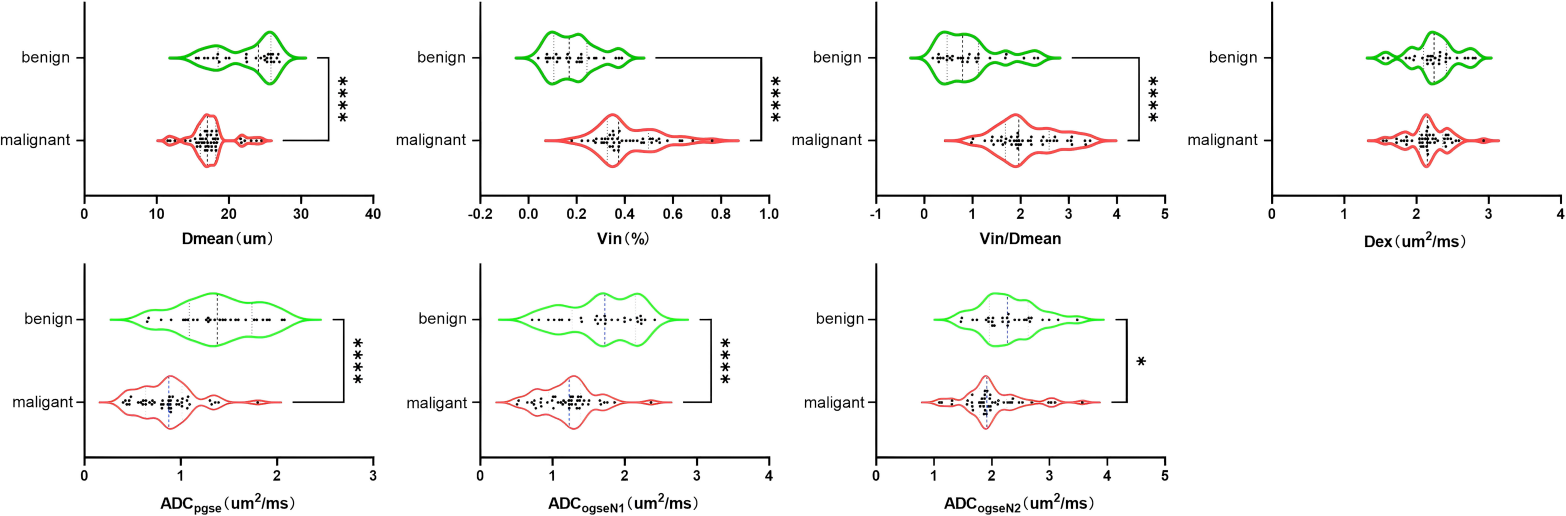
Group comparison of microstructural properties between Benign and malignant tumors of the breast.

The Intraclass Correlation Coefficient (ICC) for the delineation of breast tumor ROI by the two radiologists was 0.96 ± 0.17, p < 0.01, demonstrating excellent consistency. Various histopathological types of benign tumors and different ER/PR, Her-2, Ki-67, and LN metastasis statuses of malignant tumors were observed in the microstructural mapping derived from TD-MRI (**Figure 2**). Appendix S1 presents the diffusion signal intensity under different diffusion times. A significantly decreased mean cell diameter (Dmean) was found in malignant breast tumors compared to those in benign breast tumors (17.37 ± 2.74 µm *vs.* 22.47 ± 3.85µm, p<0.0001) as derived from OGSE and PGSE in TD-MRI. Additionally, a significantly higher intracellular volume fraction (Vin) was observed in malignant breast tumors compared to benign ones (0.41 ± 0.13% *vs.* 0.19 ± 0.10%, p<0.0001), and Vin/Dmean was also higher in malignant tumors (2.13 ± 0.66 *vs.* 0.93 ± 0.61, p<0.0001). However, there was no significant difference in extracellular diffusivity (Dex) between the two groups (p>0.05) (**Figure 3**, **Table 3**). The ADC_pgse_, ADC_ogseN1_ and ADC_ogseN2_ of malignant breast tumors were significantly lower than those of benign breast tumors (ADC_pgse_, p<0.0001; ADC_ogseN1_, p<0.0001; ADC_ogseN2_, p<0.05).

**Table 3:** Group comparison of microstructural properties between benign and malignant tumors of the breast.

	Microstructural Measures				ADC		
	Dmean(μm)	Vin(%)	Vin/Dmean	Dex(um^2/ms)	PGSE(um^2/ms)	OGSE 17Hz(um^2/ms)	OGSE 33Hz(um^2/ms)
**Malignant**	17.37±2.74	0.41±0.13	2.13±0.63	2.15±0.28	0.81±0.29	1.20±0.38	2.02±0.51
**Benign**	22.47±3.85	0.19±0.10	0.93±0.61	2.25±0.31	1.39±0.40	1.68±0.49	2.29±0.49
**P Value**	<0.0001	<0.0001	<0.0001	>0.5	<0.0001	<0.0001	<0.05

Differences between microstructural measures and ADC values of Benign and Malignant breast tumors.

**Figure 4:**
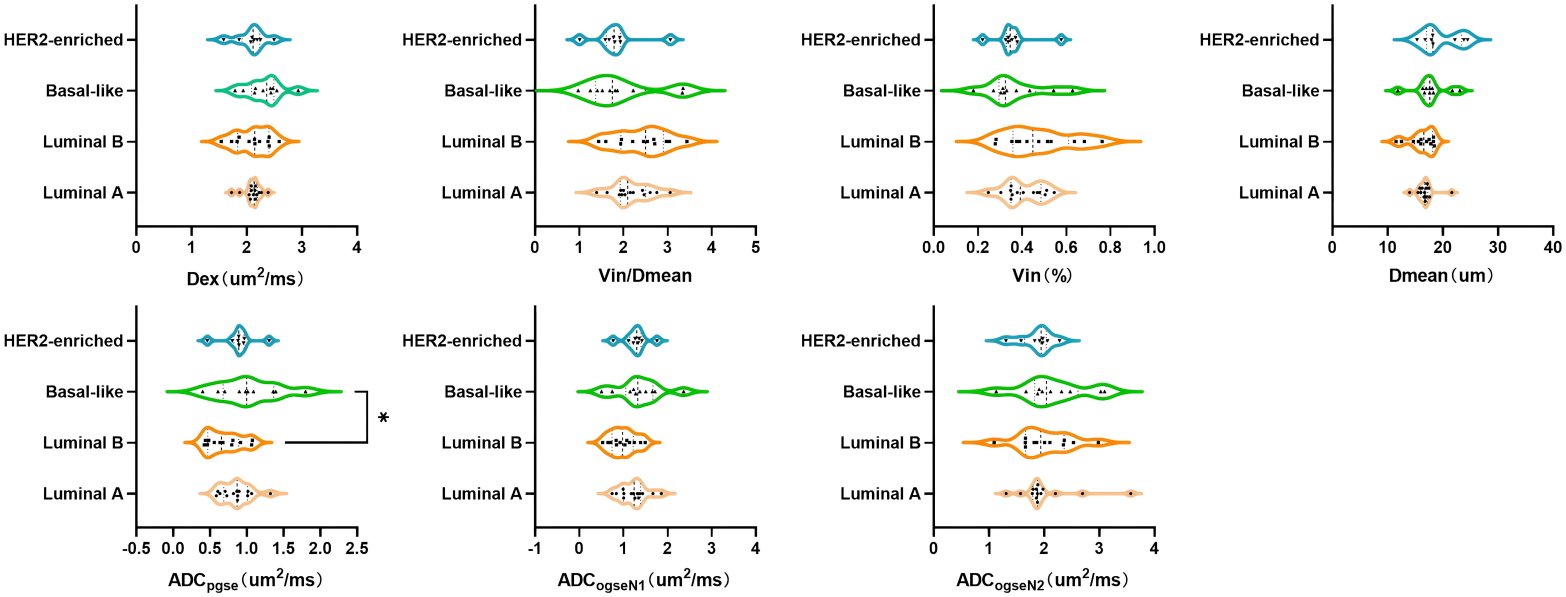
Group comparisons of microstructural properties between different molecular subtypes of breast cancer.

Among breast cancer subtypes classified by immunohistochemical receptor status, no significant intergroup differences were observed in microstructural parameters of malignant breast tumors, including Dmean, Vin Vin/Dmean, Dex (**Figure 4**). However, significant differences in the ADC with multi-b-value PGSE were identified between Luminal B and Basal-like subtypes (p < 0.05).

**Figure 5:**
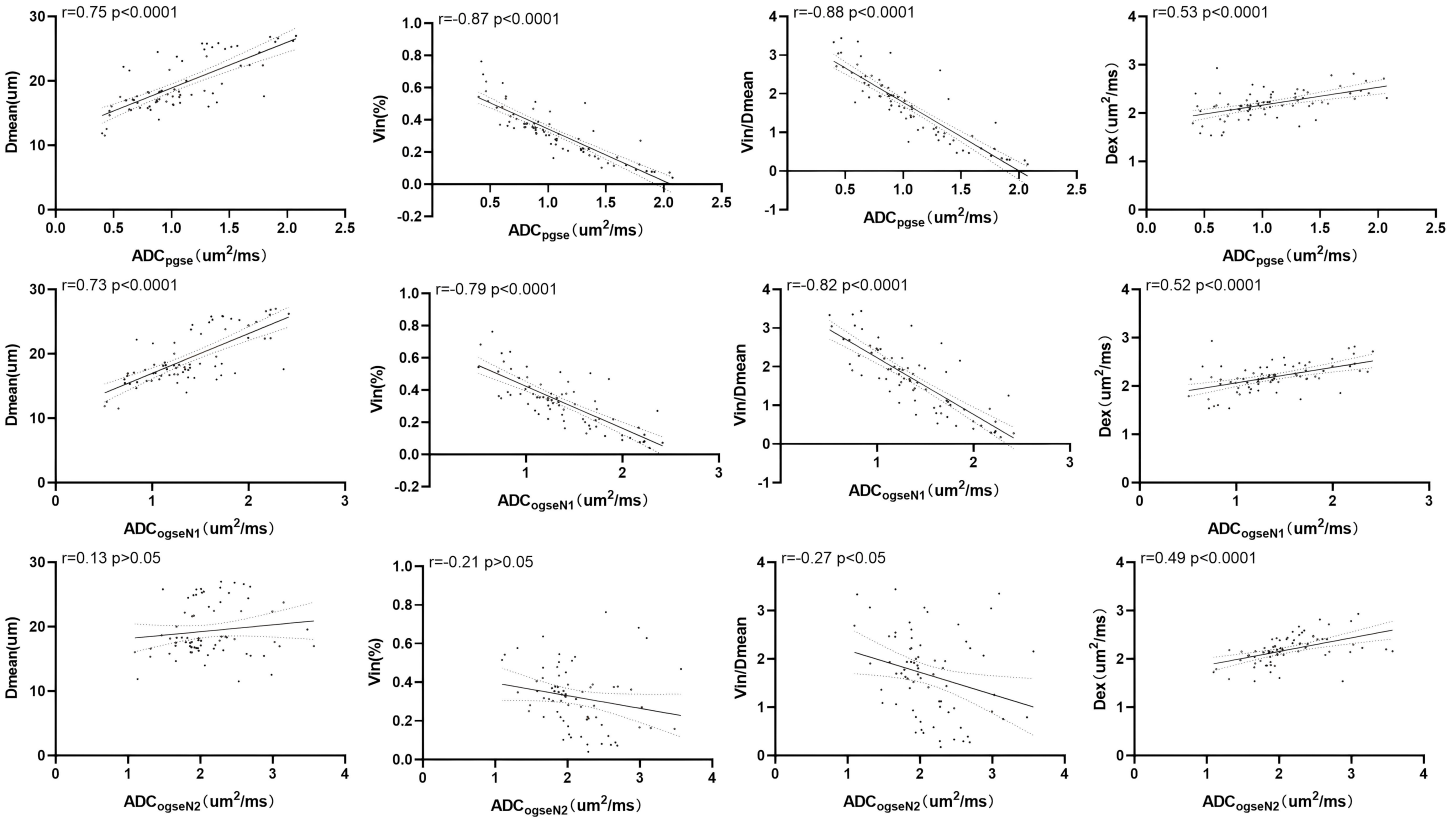
Correlation of microstructural properties with ADC values including ADCpgse, ADCogseN1, ADCogseN2.

Significant correlations were found among ADC values (including ADC_pgse_ and ADC_ogseN1_) with Dmean, Vin, and Vin/Dmean. Specifically, Dmean showed a strong positive correlation with ADC_pgse_ and ADC_ogseN1_ values (r=0.75 and r=0.73, p<0.0001), while Vin and Vin/Dmean demonstrated highly negative correlations with ADC_pgse_ and ADC_ogseN1_ (Vin: r=-0.87 and r=-0.79, p<0.0001; Vin/Dmean: r=-0.88 and r=-0.82, p<0.0001). However, Dex did not show significant correlations with ADC_pgse_ and ADC_ogseN1_ values (r=0.53 and r=0.52, p<0.0001). Furthermore, the correlation coefficients of Dmean, Vin, Vin/Dmean, and Dex with ADC_ogseN2_ were approximately 0.13 (p>0.05), -0.21 (p>0.05), -0.27 (p<0.05), and 0.49 (p<0.0001), respectively (**Figure 5**). These results indicate that ADC values are significantly influenced by Dmean and Vin, while the impact of Dex is comparatively minor.

**Figure 6:**
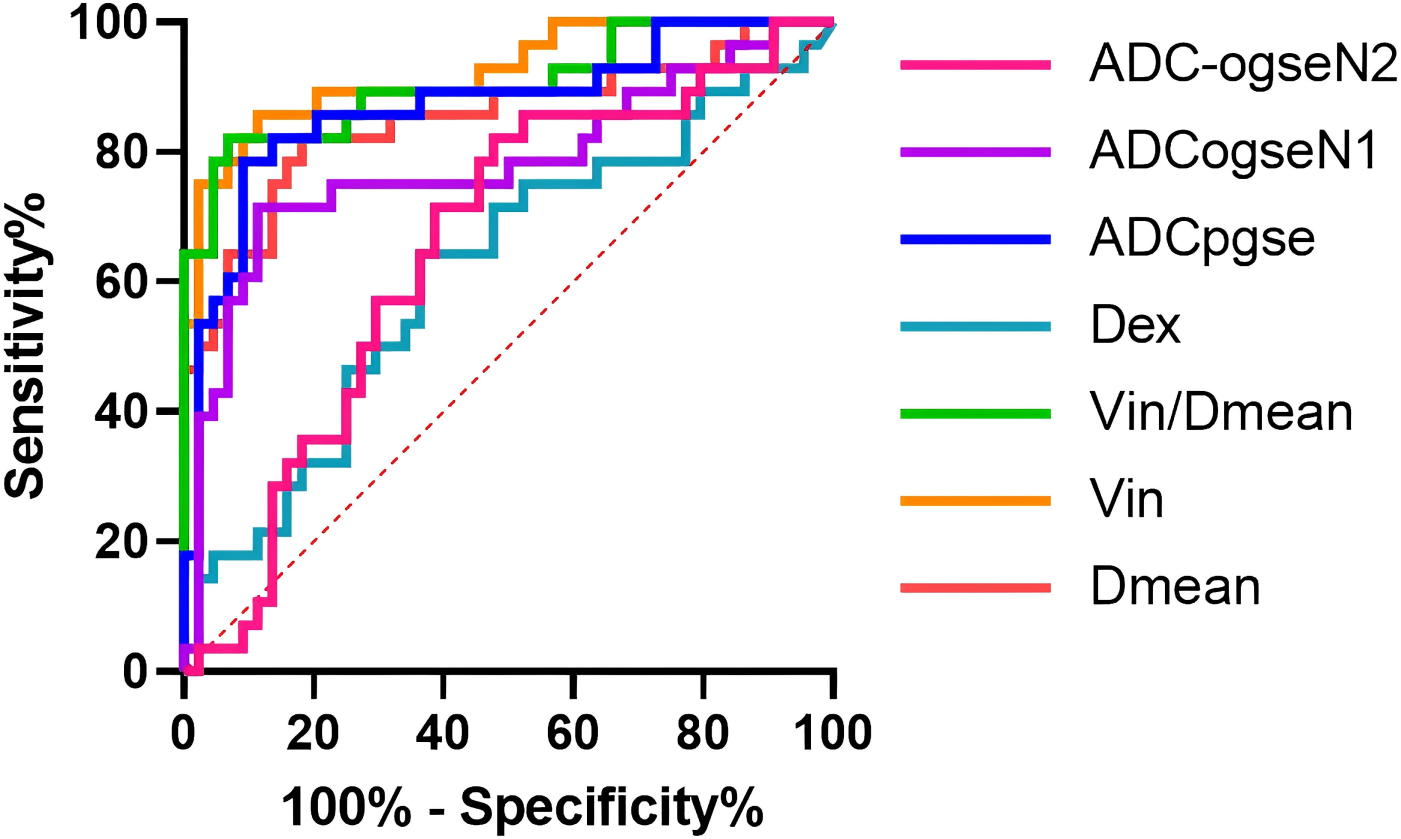
ROC curves of microstructural properties for benign–malignant differentiation of breast tumors.

Compared with ADC values, Dmean, Vin and Vin/Dmean demonstrated superior diagnostic performance in distinguishing between benign and malignant breast tumors, with the following AUC values: Dmean=0.85 (95% CI: 0.75-0.95), Vin=0.92 (95% CI: 0.86-0.997), and Vin/Dmean = 0.91 (95% CI: 0.83-0.98). In contrast, Dex showed an AUC value of 0.62 (95% CI: 0.48-0.75) (**Figure 6**, **Table 4**). The AUC values for ADC_pgse_, ADC_ogseN1_ and ADC_ogseN2_ were 0.86 (95% CI: 0.77, 0.95), 0.77 (95% CI: 0.65, 0.89), and 0.69 (95% CI: 0.56, 0.81), respectively.

**Table 4:** Diagnostic performance of time–dependent diffusion mri–derived microstructural parameters between benign and malignant tumors of the breast.

Parameter	AUC	Sensitivity	Specificity	P value
**Dmean**	0.85(0.75,0.95)	82.1%	81.8%	<0.0001
**Vin**	0.92(0.86,0.99)	85.7%	88.6%	<0.0001
**Vin/Dmean**	0.91(0.83,0.98)	82.1%	93.1%	<0.0001
**Dex**	0.62(0.48,0.75)	64.3%	63.6%	>0.5
**ADC_pgse_**	0.86(0.77,0.95)	85.7%	81.4%	<0.0001
**ADC_ogseN1_**	0.77(0.65,0.89)	67.9%	86.0%	<0.0001
**ADC_ogseN2_**	0.69(0.56,0.81)	75.0%	65.1%	<0.01

## Discussion

This study focuses on the use of TD-MRI in differentiating between benign and malignant breast tumors. With a larger sample size, decreased Dmean and elevated Vin and Vin/Dmean were observed in malignant breast tumors. The diagnostic efficacy of Dmean, Vin, and Vin/Dmean in distinguishing benign from malignant breast tumors was higher than that of ADC. Furthermore, no significant correlation was found between microscopic structural indices and different molecular subtypes of breast cancer.

Compared to Xu’s study (8), our research validated the diagnostic accuracy of OGSE in distinguishing between benign and malignant breast tumors using the histopathology as the gold standard. Moreover, our study careful delineated regions of interest (ROIs) in breast tumors, excluding areas of necrosis and cystic changes, which enabled more precise measurement of microstructural data. Ba’s (9) study focused only on malignant breast tumors, whereas our research included both benign and malignant cases, further evaluating the application of TD-MRI in breast tumors. Additionally, the inclusion criteria specifying breast tumor greater than 10 mm was based on previous studies (21) investigating various IHC statuses and LN statuses in breast cancer. This criterion was also ensured differentiation from benign breast nodules and to facilitate accurate lesion measurement in DWI sequences.

Due to the rapid growth and high heterogeneity of malignant breast tumor cells, the Dmean of malignant breast tumors is lower than that of benign tumors. However, during actual pathological processes, the active division of malignant cells frequently leads to morphological abnormalities, including: cellular volume differentiation (smaller daughter cells generated through division or abnormally enlarged multinucleated giant cells) and irregular geometric configurations (spindle-shaped or irregular polygonal forms) (26, 27). The current IMPULSED model operates under the theoretical assumption of “spherical cells with uniform distribution,” representing an oversimplification that exhibits marked deviation from the actual microstructure of malignant tumors, while the Dmean as a mean value may merely represent a rough approximation. These unaddressed factors may potentially introduce systematic deviations in Dmean measurements. Although the Dmean of benign breast tumors in this study was higher than that of malignant tumors, the Dmean values of malignant breast tumors measured here appeared slightly elevated compared to prior studies (9). This discrepancy likely arises from our exclusion of necrotic tumor components and vascular structures during region of interest (ROI) delineation. The intracellular volume fraction (Vin) reflects cell density within solid tumor cells (28). The extracellular matrix (ECM), a fundamental component of all tissues and organs, is essential for multicellular organisms. In cancer, ECM alterations can promote tumor cell growth, and extracellular diffusivity (Dex) can quantify the ECM (29, 30). This explains why the Vin of malignant breast tumors is higher than that of benign tumors.

Currently, the clinical method for distinguishing between benign and malignant breast tumors mainly relies on ADC (10, 31). In our study, ADC values (particularly ADCpgse) were significantly lower in malignant breast tumors compared to benign ones, consistent with previous findings (10, 15). However, when comparing the diagnostic performance of ADC with microstructural data from TD–MRI, Vin (AUC = 0.92), and Vin/Dmean (AUC = 0.91) exhibited higher AUC values than ADC. This discrepancy can be attributed to the influence of tumor cell size, density, and transmembrane water mobility on ADC values, factors minimized in the microstructural data derived from TD–MRI. Among the microstructural data, Dmean showed superior diagnostic performance in distinguishing between benign and malignant breast tumors.

Different breast cancer subtypes, determined by immunohistochemical receptor status, exhibit varying cell densities, vascular distributions, and invasive capabilities (32). In our study, no significant differences in microstructural characteristics were observed between different breast cancer subtypes, which is contrary to the findings reported by Wang (33). Although differences in ADCpgse were observed between Luminal B and Basal–like subtypes in our study, these findings remain inconclusive due to the limited sample size. This is different from the results of a meta–analysis including 2990 breast tumors, where ADC could not be used for molecular subtypes of breast cancer in their study (34). In other studies investigating hormone receptor expression status in breast cancer, Kim SH et al. (35) demonstrated no statistically significant differences in ADC values based on hormone receptor expression status, HER–2 status, or lymph node metastasis. However, some studies have indicated associations between ADC values and hormone receptor status (36–38). One study found higher ADC values in HER–2 positive breast cancer (39), and another showed a correlation between ADC values and lymph node metastasis in invasive ductal carcinoma (40). Therefore, these findings require further investigation with a larger sample size.

The “Cellularity” parameter derived from TD–MRI is calculated as Vin/Dmean. In Wu’s study on prostate cancer, Cellularity demonstrated the highest diagnostic performance in distinguishing prostate cancer from clinically insignificant prostate cancer (20). In Wang’s study on predicting the effectiveness of neoadjuvant therapy in breast cancer, Cellularity had the highest AUC value among all TD–MRI parameters (34). However, this parameter has not yet been widely recognized by scientists. Although this definition differs from the traditional concept of cellularity in pathology (which refers to the number of cell cross–sections per unit area on histological slides), we believe it holds potential within the TD–MRI framework and will further explore its clinical significance in future studies. In the patients included in our study, the sample sizes for certain subtypes, such as ductal intraductal papilloma, ductal carcinoma *in situ*, and lobular carcinoma *in situ*, were small. As a result, we were unable to perform a detailed analysis of the differences in microstructural parameters between the various pathological subtypes. In future studies, we plan to increase the sample size to further investigate this aspect. It is noteworthy that in cases of fibroadenoma and fibroadenomatous hyperplasia, we observed a weak dependency of diffusion signal decay on diffusion time (Appendix S1), resulting in overestimated fitted cell sizes (highlighted in red in **Figure 2**). This phenomenon may be attributed to the unique histopathological features of these lesions: fibroadenomas are predominantly composed of extracellular matrix components and exhibit relatively low cellular density, which may diminish the sensitivity of diffusion time dependency to intracellular restrictions.

There are several limitations in this study. Firstly, the selection bias due to our inclusion criteria has led to a limited number of positive cases in different subtypes of malignant breast tumors. Additionally, the number of patients with malignant breast tumors was significantly higher than those with benign tumors. This phenomenon is due to many benign breast tumor patients opting only for breast ultrasound or mammography. Additionally, some patients with benign tumors did not undergo tumor resection or biopsy, resulting in a lack of pathological data. This may hinder the identification of significant intergroup differences in microstructural data when comparing breast tumors with different immunophenotypes, contrary to findings in some previous studies. Secondly, being a single–center study, our results may not fully generalize to broader populations due to variations in patient demographics and clinical practices across different centers. Thirdly, the use of a combination of biopsy and surgical pathology results in our study poses another limitation. Not all patients with breast tumors underwent surgical excision, especially those with clinically insignificant diseases (such as benign breast tumors confirmed by biopsy and showing no changes over time) or advanced breast cancer. This variation in tissue sampling methods could lead to differences in the microstructural characteristics of tumor tissues. Finally, relative slow acquisition speed lead to only 5 slices obtained in this study. This is because the multi–b–value scanning was used to ensure accurate fitting and achieve sufficient signal–to–noise ratio. Meanwhile, relatively low TD–MRI image resolution leads to the need of other imaging modalities to identify the tumor localization. In future clinical applications, the number of b–value can be reduced to shorten scanning time.

In summary, TD–MRI has been proven with significant advantage over ADC in distinguishing between benign and malignant breast tumors in this study. In future, multi–center studies could be achieved to explore microstructural differences among tumors of different pathological types to enhance our understanding and clinical applications.

## Data Availability

The original contributions presented in the study are included in the article/**Supplementary Material**. Further inquiries can be directed to the corresponding author.
